# Categorization of the Aqueous Deficient Dry Eye by a Cut-Off Criterion of TMH Measured with Tearscope

**DOI:** 10.3390/life12122007

**Published:** 2022-12-02

**Authors:** Belen Sabucedo-Villamarin, Hugo Pena-Verdeal, Jacobo Garcia-Queiruga, Maria Jesus Giraldez, Carlos Garcia-Resua, Eva Yebra-Pimentel

**Affiliations:** Departamento de Física Aplicada (Área de Optometría), Facultade de Óptica e Optometría, Campus Vida s/n, Universidade de Santiago de Compostela, 15782 Santiago de Compostela, Spain

**Keywords:** ADDE, TMH, Tearscope, cut-off value, Tear Film and Ocular Surface Society in the second Dry Eye Workshop (TFOS DEWS-II)

## Abstract

A decrease of the Tear Meniscus Height (TMH) has been proposed as a useful indicator for Aqueous Deficient Dry Eye (ADDE) categorization. The present study aimed to calculate a TMH cut-off criterion for the categorization or severity assessment of ADDE with the Tearscope. 200 participants with a previous Dry Eye Disease (DED) diagnosis according to TFOS DEWS-II criteria were recruited. TMH by slit-lamp illumination and Lipid Layer Pattern (LLP) with Tearscope were assessed to categorise the participants into the ADDE or the Evaporative Dry Eye (EDE) group. The ADDE group was also subdivided into Mild-moderate ADDE and Moderate-severe ADDE based on TMH with slit-lamp. Additionally, the TMH was measured by Tearscope (TMH-Tc). Receiver Operating Characteristics showed that the TMH-Tc have a diagnostic capability to differentiate between ADDE and EDE participants, and between Mild-moderate or Moderate-severe ADDE, with a cut-off value of 0.159 mm (AUC = 0.843 ± 0.035, *p* < 0.001; sensitivity: 86.4%; specificity: 75.4%) and 0.105 mm (AUC = 0.953 ± 0.025, *p* < 0.001; sensitivity: 98.1%; specificity: 80.0%), respectively. The present study proposed a cut-off criterion to differentiate between ADDE and EDE participants, or between ADDE severities through TMH assessed by Tearscope.

## 1. Introduction

The tear film plays an essential role in ocular surface maintenance, providing the first line of defence and a smooth optical surface [1,2,3,4]. Dry eye is considered by many practitioners to be the most common symptom of eye discomfort in daily clinical practice. The Tear Film and Ocular Surface Society in the second Dry Eye Workshop (TFOS DEWS-II) defined Dry Eye Disease (DED) as a chronic multifactorial condition characterized by a loss of homeostasis and instability of the tear film, changing its composition, and causing ocular surface inflammation [1,3]. There are two main types of DED: Aqueous Deficient Dry Eye (ADDE) and Evaporative Dry Eye (EDE). The ADDE subtype occurs due to a reduced volume of aqueous production of the lacrimal gland, whereas EDE is usually caused by Meibomian Gland Dysfunction (MGD) or eyelid margin problems [1,5]. Therefore, an additional method for differentiation between ADDE and EDE is the assessment of the tear volume. The total tear film volume is usually evaluated indirectly through the measurement of the lower Tear Meniscus Height (TMH) since it has been estimated that the tear meniscus holds more than the 75% of the total tear film volume [1,3]. Therefore, the TMH measurement provides and specific tool for ADDE detection and thus, the assessment of ADDE severity [3].

The assessment of the TMH is universally performed by eye care practitioners with the slit-lamp biomicroscope during routine ocular examination [3,6,7]. TMH offers a valuable clinical sign for tear film volume analysis; however, there is not an accepted gold standard for the measurement method, which could lead to different values. This measurement could be performed with a millimetre reticule (directly during the eye examination) or by capturing an image of the meniscus and then processing it with image analysis software, such as ImageJ [8,9]. Other techniques, such as Optical Coherence Tomography (OCT), interferometry devices attached to the slit-lamp or multidiagnostic platforms, are used to measure TMH [10,11,12,13]. The interferometry technique is also performed with the slit-lamp biomicroscope, but using the interferometer illumination, measuring the TMH by capturing an image of the meniscus and analysing it with computer software [14,15].

The mean values of TMH in eyes without DED have been reported between 0.25 ± 0.08 and 0.29 ± 0.13 mm using the slit-lamp biomicroscope technique, between 0.19 ± 0.02 and 0.34 ± 0.15 mm with OCT and between 0.27 ± 0.12 and 0.29 ± 0.04 mm with other multidiagnostic platforms [8,12,16,17,18,19]. The TFOS DEWS-II Diagnostic Subcommittee proposed the TMH as a differential factor for the subclassification of DED, establishing a cut-off value of 0.20 mm or lower as an indicator of ADDE measured only by the slit-lamp biomicroscope technique [3,8]. Currently, studies proposed the use of innovative devices or technologies with proven repeatability and reliability, which are portable and inexpensive, to enhance the diagnosis. One such example is the Tearscope, which is one of the most widely used interferometric devices [14,15,20]. Therefore, the use of these devices creates the need to establish reliable diagnostic criteria available to practitioners. The present study aimed to establish TMH cut-off criteria for diagnosis and severity assessment of ADDE using the Tearscope.

## 2. Materials and Methods

### 2.1. Sample

A total of 200 participants (mean age of 42.84 ± 16.86 years) who attended the Optometry Clinic with an Ocular Surface Disease Index (OSDI) questionnaire score greater than 13 points were recruited for the study [21]. Only data from one eye (right eye) were included in the statistical analysis [22]. No participant was using any kind of medication or artificial tears at the time of the study. Participants were excluded if they had a prior history of ocular surgery (including refractive surgery or eyelid tattooing), ocular infections, MGD, blepharitis, glaucoma, systemic diseases, autoimmune disease, or diabetes mellitus, were pregnant or breast-feeding or wore contact lenses. All the participants gave their written informed consent to be included in the study and all procedures followed were in accordance with the Helsinki Declaration. The study protocol was approved by the Bioethics Committee of the university (USC-08/2021).

### 2.2. Study Design and Diagnostic Criteria

A battery of clinical procedures was conducted in all participants according to the TFOS DEWS-II Diagnostic Methodology report [3]. All these measurements were performed and recorded by the same examiner in a single session to avoid interobserver or intersession variability [23]. The protocol was made under controlled environmental conditions of light, temperature (20–23 °C) and humidity (50–60%). Tests were always performed in the same order, from the least to the most invasive [3]: tear film osmolarity, TMH with the slit-lamp (TMH-SL) and Tearscope (TMH-Tc), Lipid Layer Pattern (LLP), Fluorescein Break-Up Time (FBUT) and corneal staining. First, the diagnostic tests cut-off values for the DED diagnosis employed were a tear osmolarity ≥ 308 mOsm/L, an FBUT < 10 s and/or corneal staining (Oxford Scheme) ≥ 2 [3]. Second, each participant was classified according to type, as follows [1,3]:

- ADDE type: participants presented a TMH-SL ≤ 0.20 mm and an LLP > Closed Meshwork. The participants who formed the ADDE group were also subclassified into Mild-moderate ADDE if the TMH-SL was ≥0.10 mm or Moderate-severe ADDE if the TMH-SL was ≤0.10 mm.
- EDE type: participants presented a THM-SL ≥ 0.20 mm and an LLP ≤ Closed Meshwork.

According to those criteria, the sample was divided into two main groups for the analysis: ADDE and EDE group; participants classified as mixed type (TMH-SL ≤ 0.20 mm and LLP ≤ Closed Meshwork) were not included in the analysis to avoid interferences on the results [3].

### 2.3. Evaluation Procedures

#### 2.3.1. Symptomatology Assessment

DED symptomatology has been quantified by the OSDI questionary, which was self-administered by scanning a QR code provided before the examination [3,21,24]. This disease-specific questionnaire includes 12 questions asked concerning a 1-week recall period. The OSDI scores obtained were evaluated by the examiner according to the standardized guidelines on a scale of 0 to 100 points, where higher scores represent greater disability [3,21,24].

#### 2.3.2. Tear Film Osmolarity

The TearLab osmometer (TearLab Corp, San Diego, CA, USA) was used to measure the tear film osmolarity [25]. The participants were requested to sit with their heads tilted and their eyes looking at the ceiling. Then, the instrument probe was placed on the lower tear meniscus of the right eye until a beep was emitted, signalling that the sample was collected [26,27]. The TearLab converts the electrical impedance of the sample into osmolarity (mOsm/L), which was displayed on the device screen. The device measurement range extends from 275 to 400 mOsm/L.

#### 2.3.3. Tear Meniscus Height

**Figure 1:**
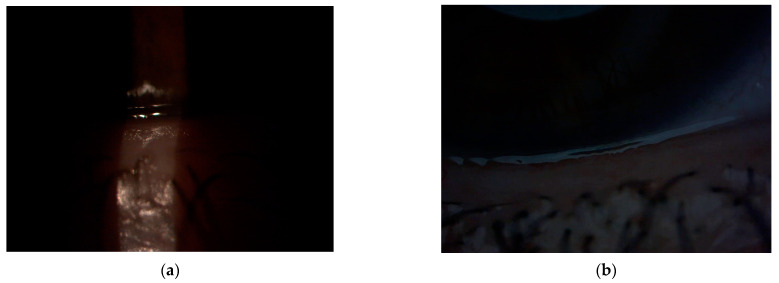
Lower tear meniscus. (**a**) Tear meniscus performed with the slit-lamp illumination; (**b**) Tear meniscus performed with the Tearscope illumination.

The TMH was measured by two methods: first, with the slit-lamp technique, and second, with an interferometer of cool white fluorescent light (Tearscope, Keeler, Windsor, UK). In both cases, the participants were properly positioned at the slit-lamp Topcon SL-D4 (Topcon Corporation, Tokyo, Japan) chin rest and instructed to look at a target to maintain primary eye gaze while the lower tear meniscus was observed with a natural blink. In both cases, the meniscus was recorded in video by the Topcon DC-4 (Topcon Corporation, Japan) digital camera attached to the slit-lamp and connected to a computer via the Topcon IMAGEnet i-base software (Topcon Corporation, Japan). The central lower meniscus was video captured at 6 o’clock position with the biomicroscope set at 0° and a 40× magnification in both techniques (Figure 1). From the least to the most invasive [3], the TMH was first assessed with the slit-lamp with 3 mm wide and 5 mm height and moderate illumination to avoid reflex tearing (Figure 1a) [8,15]. The TMH was then evaluated with the slit-lamp light off and interferometer Tearscope attached to the Topcon SL-D4 slit-lamp to keep the distance between the chinrest and the device constant during the measurement; in all TMH-Tc recordings, the brighter illumination of the Tearscope was used for better visualization (Figure 1b) [8,10,28].

The TMH images were extracted from the recorded videos and measured by a second masked observer with the open-source software ImageJ v1.53t (National Institutes of Health, Bethesda, MD, USA; http://imagej.nih.gov/ij/ (accessed on 18 January 2022)). Before the present study and similar to a previous study [15], a millimetre in a rule positioned at the same optical plane of the patients’ eye was captured and measured with the ImageJ software, giving a length of 300 pixels; this pre-study data was used to convert the pixels provided by the software to estimate the TMHs in millimetres (300 pixels = 1 mm) for further statistical analysis.

#### 2.3.4. Lipid Layer Pattern Evaluation

The LLP was evaluated with the Tearscope interferometer, which provides a visualisation of the interference pattern created by the lipid layer [23,29]. To acquire a centred visualisation while the LLP spreads completely, the Tearscope was attached to the slit-lamp and the participants were instructed to blink three times naturally without squeezing and to focus their sight straight to the centre of the device. This process was repeated three times on each participant. Once videos were recorded, images were assessed by a second masked observer. Each image was captured immediately after blinking when the lipid layer was completely expanded for one second [23]. The images were then classified following Guillon’s Scheme, a five-grade scale described as an Open Meshwork, Closed Meshwork, Wave, Amorphous and Colour LLP, that are related to thin (open and closed meshwork pattern), average (wave pattern) or thick (amorphous, and colour patterns) tear film lipid layers [23,29].

#### 2.3.5. Fluorescein Break-Up Time and Corneal Staining

The FBUT and the corneal staining measurements were both evaluated with the Keratograph 5M (Oculus Optikgerate GmbH, Wetzlar, Germany) with the participants properly positioned in the device [13,30]. Participants were instructed to look up to apply a hydrated fluorescein strip in the inferior fornix with saline solution [31].

The subjects were then requested to blink several times to ensure the adequate mixing of the dye. The FBUT was evaluated and recorded using the fluorescein function provided by the device. The FBUT was defined as the time interval between the last blink and the appearance of the first black spot on the corneal surface. This procedure was repeated three times for each participant; the final values were computed based only on the two most similar measurements [32,33]. Once videos were recorded and extracted, the FBUT was calculated by a second masked observer using the VIrtualDub64 (Open software), which provides the video recorded in frames (8 frames = 1 s) to improve the temporal resolution.

Immediately after the FBUT videos were recorded, the assessment of the corneal staining was performed through the same device, with the participant seated with their head in the same position and using fluorescein dye. The participants were first instructed to look at the centre of the device in order to evaluate the possible damage to the central cornea and then look right, left, up and down to observe the entire cornea [31,34]. The video was recorded, and the corneal staining was evaluated by a second masked observer using the Oxford Scheme once the images were extracted from the video [34]. The six Oxford scheme grades (0–5) denote the severity of dry eye: mild (stage 0 or 1), moderate (stage 2 or 3) or severe (stage 4 or 5) [34].

#### 2.3.6. Statistical Analysis

SPSS statistical software v.25.0 for Windows (SPSS Inc., Chicago, IL, USA) was used for data analysis. Significance was set at a *p* ≤ 0.05 for all statistical tests. Before analysis, the normal distribution of the data was checked using the Shapiro–Wilk test [35]; Osmolarity, FBUT, TMH-SL and TMH-Tc showed a normal distribution (Shapiro–Wilk, all *p* > 0.05) whereas OSDI, Corneal Staining and LLP were not normally distributed (Shapiro–Wilk, all *p* < 0.05). The descriptive summary of data was conducted with mean with Standard Deviation (SD) for parametric parameters, median and Interquartile Range (IQR) for non-parametric parameters, and minimum and maximum values are displayed. Differences in the values obtained in each parameter between groups or subgroups were analysed with the unpaired *t*-test on parametric variables while the Mann–Whitney U test was used for non-parametric variables.

The optimal cut-off value of the TMH-Tc to distinguish between ADDE and EDE participants as well as between Mild-moderate or Moderate-severe ADDE participants was estimated with the Receiver Operating Characteristics (ROCs) procedure [36,37,38]. Each theoretical cut-off value (from the lowest to the highest value observed in the study population) was used to estimate the sensitivity and specificity of the test. Results were then graphed with the sensitivity as a function of (1-specificity). This method of representing the relationship between the putative cut-off values and the effectiveness of the test provides a convenient way for selecting the threshold that finally provides the best combination between sensitivity and specificity (the optimal cut-off value is usually chosen as the hinge point of the curve). The discrimination power of the predictive classification model proposed was provided as the value of the Area Under the Curve (AUC) ± SD in a range from 0 (no predictive) to 1 (almost perfect). In addition, both upper and lower 95% Confidence Intervals (CI) of the AUC were also provided (Mean ± 1.96 × SD). The maximum value of Youden’s J statistic was computed (J = sensitivity + specificity − 1) to choose the best cut-off numerical criterion based on each ROC curve (hinge point of the curve), since this statistic captures the highest possible performance of a diagnostic test in a single parameter where the number of false positives and false negatives is equal.

In a second reanalysis, a cross-validation analysis was performed to assess the validity of the cut-off obtained. On each analysis, an 80% random sampling was performed previous to reanalysis with the SPSS commands. The TMH-Tc variable was converted into a new dichotomous parameter based on the cut-off calculated (0 equal to negative diagnostic and 1 to positive diagnostic), and the association of this variable and the initial diagnostic based on the TFOS DEWS-II Diagnostic Methodology report was analysed with the Cramer’s V from 0 (no predictive) to 1 (perfect predictive).

## 3. Results

Descriptive statistics for all the measurements of the sample are provided in Table 1.

### 3.1. Assessment of TMH-Tc Cut-Off Values to Differentiate between ADDE and EDE Participants

Descriptive statistics for all the measurements on each subgroup are provided in Table 2. There were no statistical differences found in the Age, OSDI, Osmolarity, Corneal staining or FBUT distribution between groups (all *p* ≥ 0.090), whereas a statistical difference was found in the TMH-SL, LLP and TMH-Tc values (all *p* ≤ 0.001).

**Figure 2:**
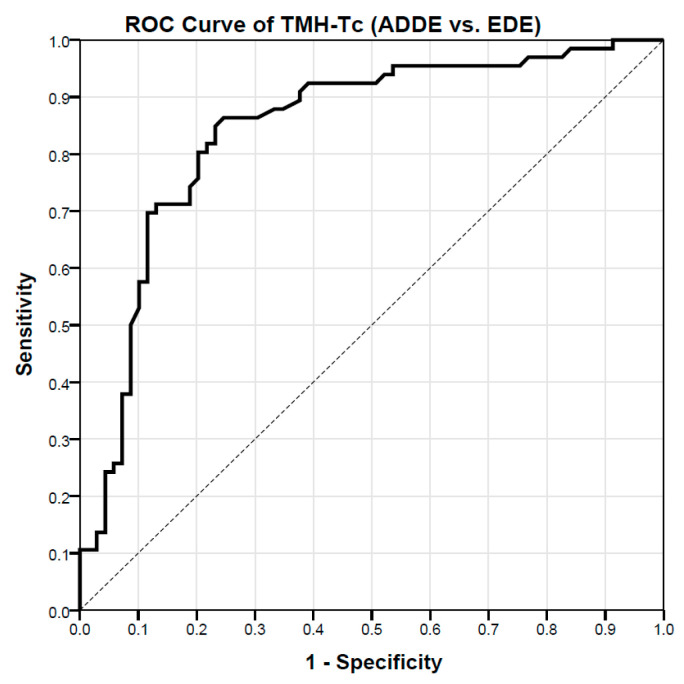
ROC curve showing the relationship between sensitivity and specificity of the TMH-Tc (ADDE vs. EDE) according to theoretical thresholds; for each of the values observed in the study population (from the lowest to the highest in either ADDE or the EDE group), the sensitivity and sensibility indexes have been calculated and reported in the graph. According to the ROC procedure (optimal cut-off value was chosen as the hinge point of the curve), the value of 0.159 mm (sensitivity: 86.4%; specificity: 75.4%) for the TMH-Tc test has been selected as the best threshold to distinguish between groups. n = 135; TMH-Tc = Tear Meniscus Height with Tearscope. ROC = Receiver Operating Characteristic; ADDE = Aqueous Deficiency Dry Eye; EDE = Evaporative Dry Eye.

ROCs procedures showed that TMH-Tc has a diagnostic capability to differentiate between participants’ types (AUC = 0.843 ± 0.0354, *p* < 0.001, 95% CI = 0.774–0.912). By calculating the Youden’s index (Youden’s J statistic = 0.617), there was found a cut-off value for the THM-Tc of 0.159 mm (sensitivity: 86.4%; specificity: 75.4%) to distinguish ADDE from EDE participants (Figure 2). The cross-validation analysis on a random sampling of 80% showed a strong association of the calculated TMH-Tc with the previous proposed diagnostic criteria of the TFOS DEWS-II to differentiate between ADDE and EDE participants (Cramer’s V = 0.657, *p* < 0.001).

### 3.2. Assessment of TMH-Tc Cut-Off Values to Differentiate between Mild-Moderate and Moderate-Severe ADDE Participants

Descriptive statistics for the ADDE Mild-moderate subgroup are provided in Table 3. There was found no statistical difference in the Age, OSDI, Osmolarity, Corneal staining or LLLP distribution between ADDE subgroups (all *p* ≥ 0.052), whereas a statistical difference was found in the FBUT, TMH-SL and TMH-Tc values (all *p* ≤ 0.047).

**Figure 3:**
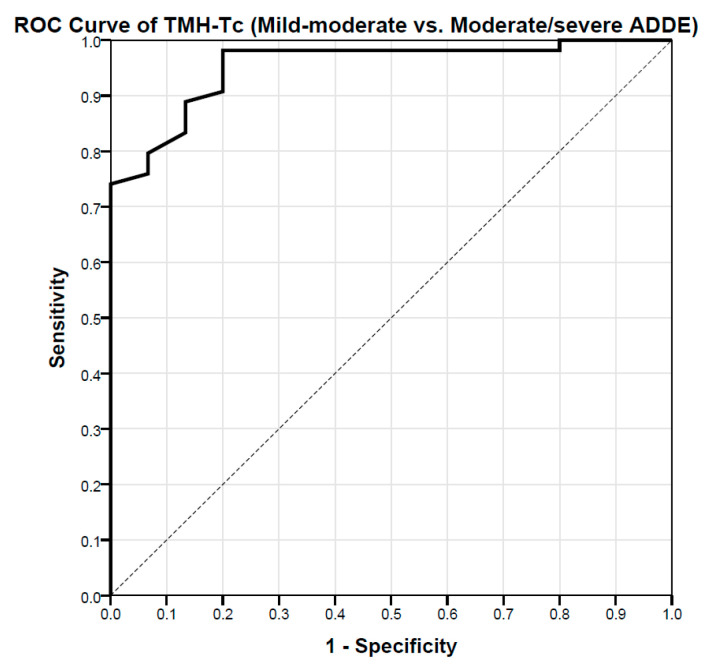
ROC curve showing the relationship between sensitivity and specificity of the TMH-Tc (Mild-moderate ADDE vs. Moderate-severe ADDE) according to theoretical thresholds; for each of the values observed in the study population (from the lowest to the highest in either Mild-moderate or the Moderate-severe group), the sensitivity and sensibility indexes have been calculated and reported in the graph. According to the ROC procedure (optimal cut-off value was chosen as the hinge point of the curve), the value of 0.105 mm (sensitivity: 98.1%; specificity: 80.0%) for the TMH-Tc test has been selected as the best threshold to distinguish between groups. n = 69. TMH-Tc = Tear Meniscus Height with Tearscope. ROC = Receiver Operating Characteristic; ADDE = Aqueous Deficiency Dry Eye.

ROCs procedures showed that TMH-Tc has a diagnostic capability to differentiate between patient severities (AUC = 0.953 ± 0.0230, *p* < 0.001, 95% CI = 0.908–0.999). By calculating the Youden’s index (Youden’s J statistic = 0.782), there was found a cut-off value for the THM-Tc of 0.105 mm (sensitivity: 98.1%; specificity: 80.0%) to distinguish Mild-moderate from Moderate-severe ADDE participants (Figure 3). The cross-validation analysis on a random sampling of 80% showed an almost perfect association of the calculated TMH-Tc against the previous proposed diagnostic criteria of the TFOS DEWS-II to differentiate Mild-moderate from Moderate-severe ADDE participants (Cramer’s V = 0.854, *p* < 0.001).

## 4. Discussion

In the actual clinical visual health sciences, there is a growing interest in the use of novel devices that are more portable and easier to use for daily routine explorations and management of DED [14,20]. The status of the tear film has great relevance for DED assessment, being that the total tear film volume is a relevant parameter for its characterisation [3,6]. TMH has been proposed as the most useful procedure to provide an indirect measurement of the tear film volume, as it is able to differentiate between the two main subgroups of DED: the ADDE and the EDE [6,39]. Over the past few decades, different non-invasive methods have been proposed to obtain the TMH measurement instead of traditional measurement with slit-lamp [12]. However, there is still the inconvenience of the high cost and size of those instruments. To shortcut those limitations, devices such as the Tearscope or Easytearview+ (Easytear S.R.L, Italy) interferometers have been introduced and chosen by practitioners [10,11,13,40]. The usefulness and capability of the Tearscope device to assess the TMH is similar to the traditional slit-lamp biomicroscope technique [20] without significant TMH measurements [14,15], and is a simple, reliable and repeatable tool that can be used for the assessment of DED patients [14,15,20].

Several authors obtained mean values of TMH in different dry eye cohort participants that go from 0.19 ± 0.05 to 0.20 ± 0.04 mm in ADDE, from 0.271 ± 0.117 to 0.29 ± 0.09 mm in MGD, from 0.203 ± 0.050 to 0.22 ± 0.09 mm in ADDE with MGD participants and 0.169 ± 0.029 mm for undefined dry eye participants [41,42,43]. In the present study, mean values of TMH, similar to previous studies, were obtained for ADDE and EDE (0.143 ± 0.054 and 0.228 ± 0.092 mm respectively), showing that ADDE participants have lower TMH values than EDE participants (Unpaired *t*-test, *p* < 0.001).

Although there are studies that compare the results obtained with the different illuminations of the devices, those authors have not sought a cutting criterion to differentiate between ADDE and EDE diagnosis, or ADDE severities assessment [11,14,15,20]. Niedernolte et al. [11] evaluated the measurement of the TMH with slit-lamp and Tearscope illumination using pre-setting cut-off values of 0.20, 0.22, and 0.24 mm, showing that the measurement with reticule assistance under 8× and 32× magnification was able to discriminate between normal and low TMH participants using these three criteria. In addition, they showed that measuring the TMH by image analysis software was unable to differentiate between normal and low TMH participants when the cut-off was set at 0.20 mm, which did not happen with the cut-off values of 0.22 or 0.24 mm [11]. According to the ROCs procedure, the optimal cut-off value is usually chosen as the hinge point of the curve between sensitivity and specificity for each of the observed values in the study population; the present study found a cut-off criterion when the measurement of the TMH was performed under Tearscope illumination and by image analysis for ADDE diagnosis of 0.159 mm, with a sensitivity and specificity of 86.4% and 75.4%, respectively. Researchers suchg as Singh et al. [43] found a cut-off value of 0.204 mm to distinguish between healthy and ADDE participants, with a sensitivity and specificity of 98.3% and 96.67%, respectively. Additionally, Qiu et al. [44] found a cut-off value of 0.248 mm to differentiate healthy from ADDE participants, with a sensitivity of 61% and a specificity of 77%. These cut-off values were slightly higher than those obtained in the present study; however, it should be noted that these reports compared healthy and DED participants (not ADDE vs. EDE participants); moreover, the device used for the TMH measurement was the OCT [11,43,44]. Furthermore, the sensitivity and specificity obtained in the present study measured the TMH using Tearscope illumination and image analysis, which is a reliable option for practitioners. OCT instruments are expensive and are not portable, and the image interpretation is more complex than the Tearscope. Measuring the TMH with slit-lamp illumination, Garcia-Resua et al. [8] showed that the mean value of the TMH in healthy participants was 0.25 ± 0.08 mm. Similar results were obtained by Imamura et al. [45], where the mean TMH values measured with slit-lamp illumination and reticule were 0.21 ± 0.50 mm. The differences between the illumination and visualization systems used in those previous studies affect the mean values of each technique, in concordance with the present study, which showed lower mean values. Furthermore, it should be considered that in the present study the resolution technique is higher, due to the use of image analysis quality that a slit-lamp with reticule does not provide [15].

Besides the detection of ADDE, the TMH could provide different degrees of severity; the TFOS DEWS-II proposed that a TMH lower than 0.10 mm may distinguish between Moderate-severe and Mild-moderate ADDE [3]. The present study proposed a possible criterion for ADDE moderate-severe in agreement with the described before when the TMH measured with Tearscope was lower than 0.105 mm (98.1% sensitivity and 80% of specificity) that could allow the clinical differentiation in ADDE subgroups. To the best of the researcher’s knowledge, no other report has proposed a cut-off criterion to differentiate between ADDE subgroups.

The main strength of the present study was the high total sample size recruited (200 participants). In addition, it is important to note that strict diagnosis and classification criteria according to the TFOS DEWS-II Diagnostic Methodology report were used [3]. In addition, the characterisation and exclusion of the DED mixed group type from the analyses to avoid interference gave more accuracy to the results, because those participants have both, the lipid phase and the tear film volume altered. On the other hand, the main limitation of the present study was the small size of the ADDE participants in the Moderate-severe subgroup, where the number of volunteers was considerably lower than in the other group; additionally, the classification of the participants in the DED subgroups was performed by using slit-lamp illumination, which implies a limitation of the study too. Another limitation is the requirement of digital imaging and the subsequent measurement required; the clinician does not get an immediate measurement that could be used in the routine clinic. In addition, the cut-off values obtained are specific for the Tearscope; while similar devices, such as the EASYTEARview, would simply act as a cold light source and would be expected to show similar cut-off criterion (the difference may only lie in the observation system) since both devices have a similar but not completely equal design (i.e., the Tearscope has a cone larger than the EASYTEARview; therefore, the distance that the illumination is generated could affect the visualization), new validation studies for those devices may be required. Those issues should be addressed in future clinical research investigations.

## 5. Conclusions

In conclusion, cut-off values to differentiate between the type of dry eye (ADDE or EDE) and between ADDE severity through the assessment of the TMH by the Tearscope have been proposed. Those proposed values could be useful to eye practitioners to implement the Tearscope, an easy-to-use portable device, in their dry eye diagnosis routinary clinical assessment. The next step must be to validate the diagnostic reliability of the cut-off values obtained in the present study in a different DED participant sample.

## Figures and Tables

**Table 1:** Descriptive statistics of all samples.

		Age (Years) *	OSDI (Score) **	Osmolarity (mOsm/L) *	Corneal Staining (Oxford Scheme) **	FBUT (s) *	TMH-SL (mm) *	LLP (Guillon Scheme) **	TMH-Tc (mm) *
Total Sample (n = 200)	Mean/Median	42.84	25.00	318.40	1.00	7.76	0.186	Close Meshwork	0.173
	SD/IQR	16.86	18.75–35.42	18.51	0.00–1.00	6.17	0.089	Open Meshwork—Wave	0.077
	Minimum	18.00	12.50	275.00	0.00	1.45	0.060	Open Meshwork	0.040
	Maximum	71.00	77.08	400.00	4.00	41.60	0.740	Colour	0.640

SD = Standard Deviation. IQR = Interquartile Range. OSDI = Ocular Surface Disease Index. FBUT = Fluorescein Break-Up Time. TMH-SL = Tear Meniscus Height with Slit-Lamp. LLP = Lipid Layer Pattern. TMH-Tc = Tear Meniscus Height with Tearscope. * Mean and SD displayed on parametric parameters. ** Median and IQR displayed on non-parametric parameters.

**Table 2:** Descriptive statistics of the ADDE and EDE groups.

		Age (Years) *	OSDI (Score) **	Osmolarity (mOsm/L) *	Corneal Staining (Oxford Scheme) **	FBUT (s) *	TMH-SL (mm) *	LLP (Guillon Scheme) **	TMH-Tc (mm) *
ADDE (n = 69)	Mean/Median	46.14	25.00	319.83	1.00	8.55	0.140	Wave	0.143
	SD/IRQ	14.95	18.75–39.58	16.51	0.00–1.50	6.17	0.037	Wave-amorphous	0.054
	Minimum	18.00	12.50	291.00	0.00	1.45	0.060	Wave	0.04
	Maximum	71.00	60.42	400.00	4.00	41.60	0.190	Colour	0.33
EDE (n = 66)	Mean/Media	42.86	22.57	312.50	1.00	7.39	0.270	Closed Meshwork	0.228
	SD/IQR	18.66	16.67–34.09	19.42	0.00–1.00	4.20	0.105	Open Meshwork—Closed Meshwork	0.092
	Minimum	18.00	12.50	275.00	0.00	1.83	0.200	Open Meshwork	0.080
	Maximum	71.00	77.08	400.00	4.00	22.75	0.740	Closed Meshwork	0.640
	*p*	0.260 ^‡^	0.142 ^†^	0.090 ^‡^	0.300 ^†^	0.300 ^‡^	<0.001 ^‡^	<0.001 ^†^	<0.001 ^‡^

SD = Standard Deviation. IQR = Interquartile Range. OSDI = Ocular Surface Disease Index. FBUT = Fluorescein Break-Up Time. TMH-SL = Tear Meniscus Height with Slit-Lamp. LLP = Lipid Layer Pattern. TMH-Tc = Tear Meniscus Height with Tearscope. * Mean and SD displayed on parametric parameters. ** Median and IQR displayed on non-parametric parameters. ^‡^ Unpaired *t*-test. ^†^ Mann–Whitney U test.

**Table 3:** Descriptive statistics of the ADDE Mild-moderate and ADDE Moderate-severe subgroups.

		Age (Years) *	OSDI (Score) **	Osmolarity (mOsm/L) *	Corneal Staining (Oxford Scheme) **	FBUT (s) *	TMH-SL (mm) *	LLP (Guillon Scheme) **	TMH-Tc (mm) *
ADDE Mild-moderate	Mean/Median	45.24	29.17	317.65	1.00	9.45	0.157	Wave	0.159
	SD/IQR	15.16	18.75–37.50	13.35	0.00–1.00	8.68	0.022	Wave—amorphous	0.050
	Minimum	18.00	12.50	291.00	0.00	1.75	0.011	Wave	0.070
(n = 64)	Maximum	68.00	60.42	368.00	3.00	14.60	0.200	Colour	0.330
ADDE Moderate-severe (n = 15)	Mean/Median	48.40	25.00	327.13	1.00	4.91	0.081	Wave	0.228
	SD/IQR	14.45	20.00–41.67	23.81	0.00–2.00	3.01	0.023	Wave-Colour	0.092
	Minimum	20.00	12.50	303.00	0.00	1.45	0.060	Wave	0.080
	Maximum	71.00	60.42	400.00	4.00	10.45	0.100	Colour	0.640
	*p*	0.513 ^‡^	0.884 ^†^	0.052 ^‡^	0.056 ^†^	0.047 ^‡^	<0.001 ^‡^	0.222 ^†^	<0.001 ^‡^

SD = Standard Deviation. IQR = Interquartile Range. OSDI = Ocular Surface Disease Index. FBUT = Fluorescein Break-Up Time. TMH-SL = Tear Meniscus Height with Slit-Lamp. LLP = Lipid Layer Pattern. TMH-Tc = Tear Meniscus Height with Tearscope. * Mean and SD displayed on parametric parameters. ** Median and IQR displayed on non-parametric parameters. ^‡^ Unpaired *t*-test. ^†^ Mann-Whitney U test.

## Data Availability

Not applicable.
